# Arts, cultural heritage, sciences, and micro-/bio-/technology: Impact of biomaterials and biocolorants from antiquity till today!

**DOI:** 10.1093/jimb/kuae049

**Published:** 2024-12-04

**Authors:** Maarten L De Mol, Erick J Vandamme

**Affiliations:** Centre for Industrial Biotechnology and Biocatalysis (InBio.be), Department of Biotechnology, Faculty of Bioscience Engineering, Ghent University, Coupure Links 653, 9000 Ghent, Belgium; Centre for Industrial Biotechnology and Biocatalysis (InBio.be), Department of Biotechnology, Faculty of Bioscience Engineering, Ghent University, Coupure Links 653, 9000 Ghent, Belgium

**Keywords:** Art, Natural Products, Biomaterials, Biocolorants

## Abstract

Nature has inspired and provided humans with ideas, concepts, and thoughts on design, art, and performance for millennia. From early societies when humankind often took shelter in caves, until today, many materials and colorants to express feelings or communicate with one another were derived from plants, animals, or microbes. In this manuscript, an overview of these natural products used in the creation of art is given, from paintings on rocks to fashionable dresses made from bacterial cellulose. Besides offering many examples of art works, the origin and application of various biomaterials and colorants are discussed. While many facets of our daily lives have changed over millennia, one certainty has been that humans have an intrinsic need to conceptualize and create to express themselves. Driven by technological advances in the past decades and in the light of global warming, new and often more sustainable materials and colorants have been discovered and implemented. The impact of art on human societies remains relevant and powerful.

**One-Sentence Summary:**

This manuscript discusses the use of biomaterials and biocolorants in art from a historical perspective, spanning 37,000 bc until today.

## Graphical Abstract

**Graphical Abstract:**
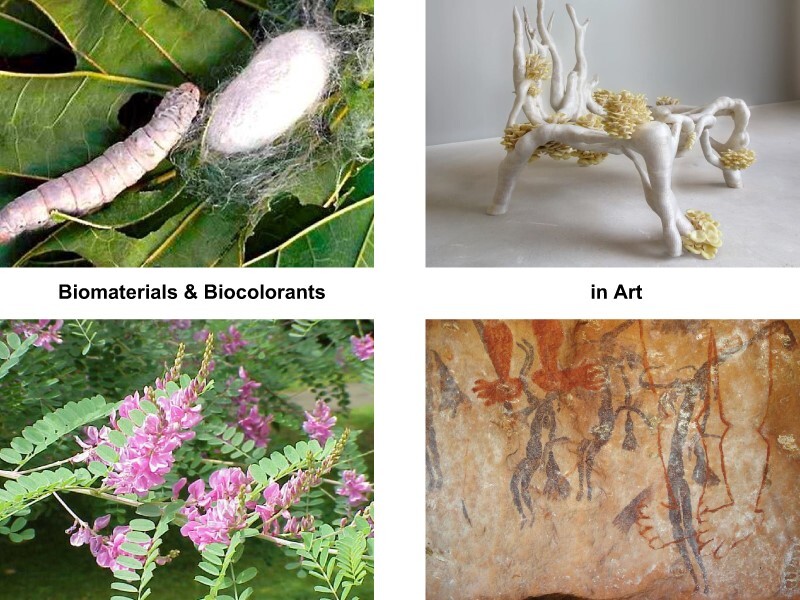

## Introduction and Definitions

Throughout history from antiquity till the present, the arts (and cultural heritage in general) and the sciences as well as (bio)technologies have always been intertwined, although largely in a hidden way. “Art” is a term difficult to define as it is the combined result of creativity, ratio, and fantasy, knowledge, design, and experience that ends up in a physical work, object, or performance with unexpected or improved esthetic value and emotion, and/or with broad social impact. It is often based on the most individual expression of the most individual emotion of the artist involved. “Artworks” relate to immovable cultural heritage (such as prehistoric cave paintings, ancient buildings, temples, pyramids, frescos, and monuments) and to movable cultural heritage (such as archives, manuscripts, collections, statues, paintings, fabric, garments, furniture, libraries, and plays). Contemporary art is “the art of today,” produced since the second half of the 20th century. These artists work in a globally influenced, culturally diverse, and technologically fast advancing world. Their art is often a dynamic combination of novel materials, methods, concepts, and subjects that challenge long-existing boundaries (Berry et al., 2002; Maloy, 2016; Schwanauer & Levitt, 1984; Snow, 1959).

Science is the result of a gradual buildup and interaction over time of verifiable human knowledge. Technology refers to the applications of knowledge and sciences for useful practical aims. Equally, even unknown to most current biotechnologists, the term “biotechnology” was already coined in 1919 by Hungarian agricultural engineer Prof. Karoly Ereky (1878–1952), University of Budapest, and was introduced in the scientific literature and in practice to describe “processes, that upgrade renewable agro- and biological resources into socially useful products” (Ereky, 1919; Fary & Kralovansky, 2006). In his vision, microbes, plants, and animals had to be seen as biochemical and technological production biosystems. The term faded away and became again in fashion only in the 1970s, now mainly focusing on recombinant DNA (rDNA), microorganisms (bacteria, yeasts, and fungi), plants, and cultured higher cells and tissues as production biosystems. Nowadays, these biotechnologies are increasingly used to develop new ways for efficient and eco-friendly production of a wide range of tailor-made biochemicals, enzymes, and biopharmaceuticals, including biopolymers, biomaterials, and colorants for food, feed, pharma, cosmetics, materials, fabrics, etc. and for artworks (Rai et al., 2021; Reade, 2023).

**Fig. 1.**
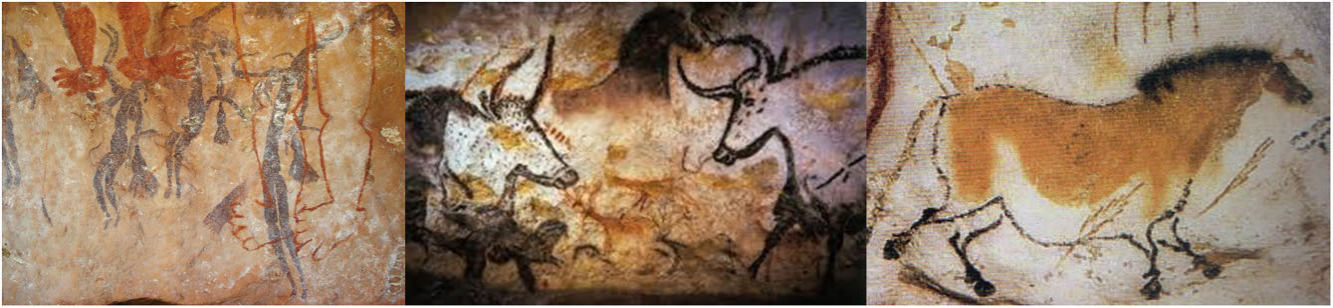
Drawings made by early humans in caves and on rock walls. From left to right: Aboriginal rock art discovered in the Gabarnmung rock site (±28,000 bc), and rock art uncovered in the Chauvet (±37,000 bc) and Lascaux caves (±18,000 bc) in France.

Interactions between the arts and the biosciences (biology, biochemistry, microbiology, fermentation, biotechnology, genetics, etc.) as well as with technology are indeed not at all new and actually started unknowingly in antiquity. Since about 37,000 bc, early modern humans depicted human figures, animals, and plants in caves and on rock walls, initially in a primitive way with main emphasis on contours, shape, and color (Fig. 1) (Leroi-Gourhan, 1982; Quiles et al., 2016). Gradually, these interactions became more sophisticated, with works of art being intentionally produced, using also biomaterials and biochemicals derived from animals (charred bone, ivory, and milk- or egg yolk-based tempera) and fiber materials (such as wool, silk, and parchment) and colorants such as insect cochineal. Such biomaterials were also derived from plants (wood, fibers such as linen, hemp, jute, cotton, cellulose, etc.), as well as plant-derived biochemicals including oils, waxes, and dyes such as blue indigotin, purple–red brazilin, yellow luteolin, and red alizarin. In many cases the relevant animals and plants were later intentionally bred or grown, respectively. Modern microbial biotechnology allows now for the synthesis of a range of biocolorants and pigments (indigo, violacein, flavins, carotenoids, quinones, etc.) or biomaterials/biopolymers (bacterial cellulose, fungal mycelium, etc.) and even bio-art on its own via metabolically and rDNA-engineered microbes and controlled fermentation processes (Albermann, 2011; Berry et al., 2002; Grewal et al., 2022; Park et al., 2021; Rai et al., 2021; Reade, 2023; Vandamme, 2019; 2021b; Verwaal et al., 2007; Walker et al., 2023, 2024; Yang et al., 2021).

Furthermore, science and (bio)technology provide now indispensable tools in identifying and verifying artworks, and also in restoration and conservation of art objects (Konkol et al., 2008). On the other hand, some (micro-) organisms (bacteria, fungi, microalgae, insects, etc.) did—and still do—also damage artworks worldwide over the years in combination with air pollution and other atmospheric conditions (Cappitelli et al., 2020; Ciferri, 1999; Dixon, 2013a; Lopez-Miras et al., 2013; Pepe et al., 2010; Rahman et al., 2016).

**Fig. 2.**
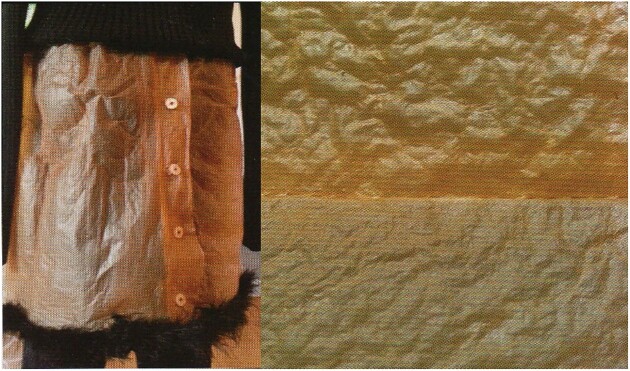
Left: A dress made of bacterial cellulose. Right: Bacterial cellulose fabric.

Current realizations, concepts, and interactions of art with science and biotechnology in its broadest meaning (encompassing biology, microbiology, biochemistry, life and agro-sciences, fermented food and drinks, etc.) are now emerging in the public domain and appeal to the general public, although such interactions are ongoing since ages, mainly unknowingly. Microbial biopolymers/biomaterials produced by bacteria and fungi form nowadays the matrix for several 3D printing materials, and fashion dresses woven with fungal mycelium and bacterial fiber-based fabrics are now in fashion (Fig. 2) (Zhong, 2020). Also, biocolorants produced by (metabolically or membrane-engineered) microbes via controlled fermentation and via plant cell cultures are now a hot topic: it all sounds very innovative and novel, the way how modern biotechnology recently impacts science and art (Grewal et al., 2022; Yang et al, 2021). However, “biotechnology avant la lettre” performed similar tasks already for over millennia (Dixon, 2013a; Finlay, 2002; Greenfield, 2005; Maloy, 2016; Vandamme, 1989, 2002, 2011, 2016; 2019, 2021a, 2021b; Vandamme et al., 1998; Vandamme & Soetaert, 2006). The following examples, focusing on the origin, characteristics, and the use of biomaterials and biocolorants (insoluble pigments and soluble colors) related to daily life but also to works of art and cultural heritage, may underpin this bold statement.

## Biopolymers and Biomaterials Derived From Plants, Animals, and Microbes: Their Use in Works of Art Over Time

### Derived From Plants

Wooden sculptures, furniture, statues, frames, paper, canvases, textiles, etc. are universally in use in society but also in the art world. They are made from a mixture of biopolymers (lignocellulosics, hemicellulose, lignin, and pectin) of plant origin (wood, cotton, bamboo, flax, papyrus, etc.). While often artworks are made up from plant-derived biopolymers and/or biomaterials, they also find applications in the conservation of national heritage or artworks through their use as gels and films (Caruso et al., 2023).

### Derived From Animals

**Fig. 3.**
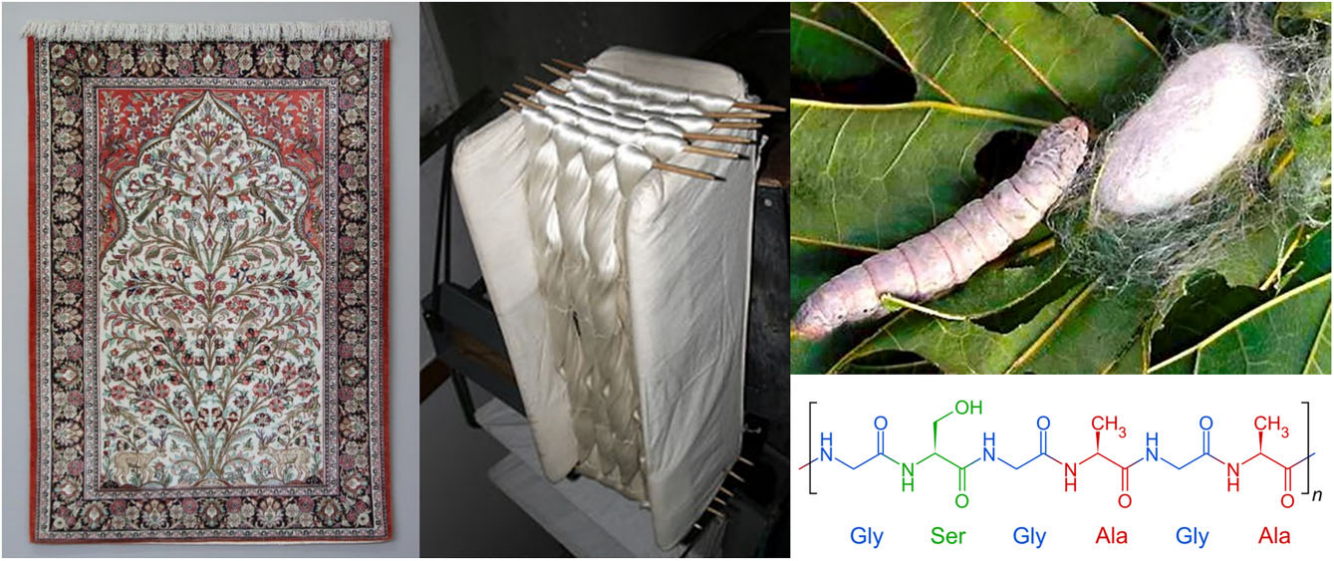
Eastern silk art tapestry and spun silk yarn (left) produced by the mulberry silkworm (top right). The silk fibroin fiber consists of a serial condensation of amino acids (bottom right).

Silk is an animal protein structure (a fiber mix of fibroin and sericin) produced by and spun around its cocoon by the mulberry silkworm (*Bombyx mori*). People collect/harvest the cocoons since centuries and weave the fiber into expensive garments and other clothing or into costly tapestry and floor carpets. Especially Persian/Eastern silk art tapestries are world famous in this context and are considered as unique artworks (Fig. 3). Also higher animals have played an indirect or direct role in ancient and recent works of art. The famous ornamental and arty ivory carvery figurines (such as the *Venus of Hohle Fels* and the *Löwenmensch* of Hohlenstein-Stadel) dating from 40,000 bc (Conrad, 2003, 2009) and ivory utensils (combs, hairpins, etc.) from teeth and tusks of animals (mammoth, elephant, walrus, whalebone, hippopotamus, etc.) have been made since antiquity on all continents. Later on, worked ivory became very popular as “inlays” material in luxury furniture and statues. Animal teeth in general are built up of dentin and enamel tissue that largely results in crystallized hydroxyapatite during teeth formation. Over the last decades, ivory trade is controversial—if not illegal— and has been banned in many countries, making ivory art even more exclusive.

**Fig. 4.**
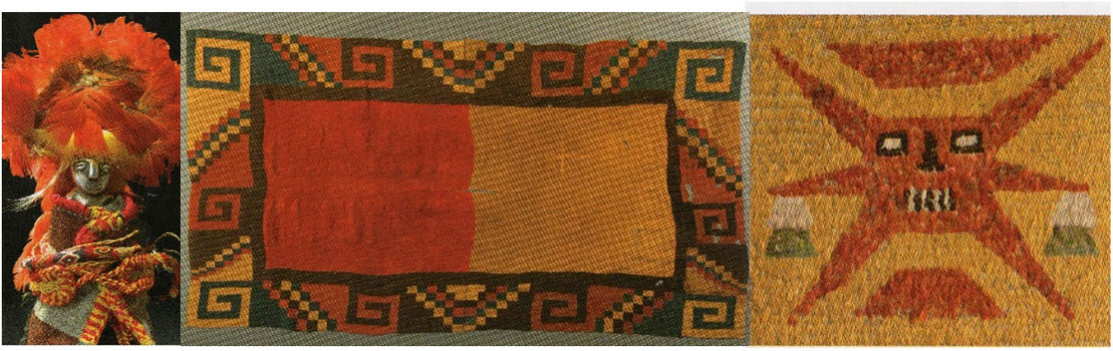
Colorful Andes artwork made with feathers and wool.

A milk or egg protein mix named “tempera” was long in use as a paint component till the ad 1500s, when plant (linseed) oils took over as fast drying components for pigments and paintings. Parchment (mainly collagen protein) is a writing or painting animal-derived “membrane” made from scraped and dried untanned skins of animals, mainly from sheep, cows, and goats. Vellum is a high-quality parchment made from the skins of young animals (lambs and calves). Both “membranes” have been used as a writing, calligraphy, and illustration medium for over four millennia and were gradually replaced by clay tablets and papyrus. Only in the 15th century they were largely replaced by paper, also in the advent of printing. They remain praised by some artists, in use for “ritual” Torah scrolls, or other important documents and became a craft and art material up till today (Ryder, 1964). In the Precolumbian Andes civilizations, wool (a keratin type of protein) from llamas, vicunas, and alpacas was a very important raw material for clothing and fabrics and was also in use for arty textiles and ornaments. The Inca emperors regarded these textiles, often brilliantly and naturally colored, as an extremely precious asset and they were a symbol of power and identity (Fig. 4) (Stone, 2012).

**Fig. 5.**
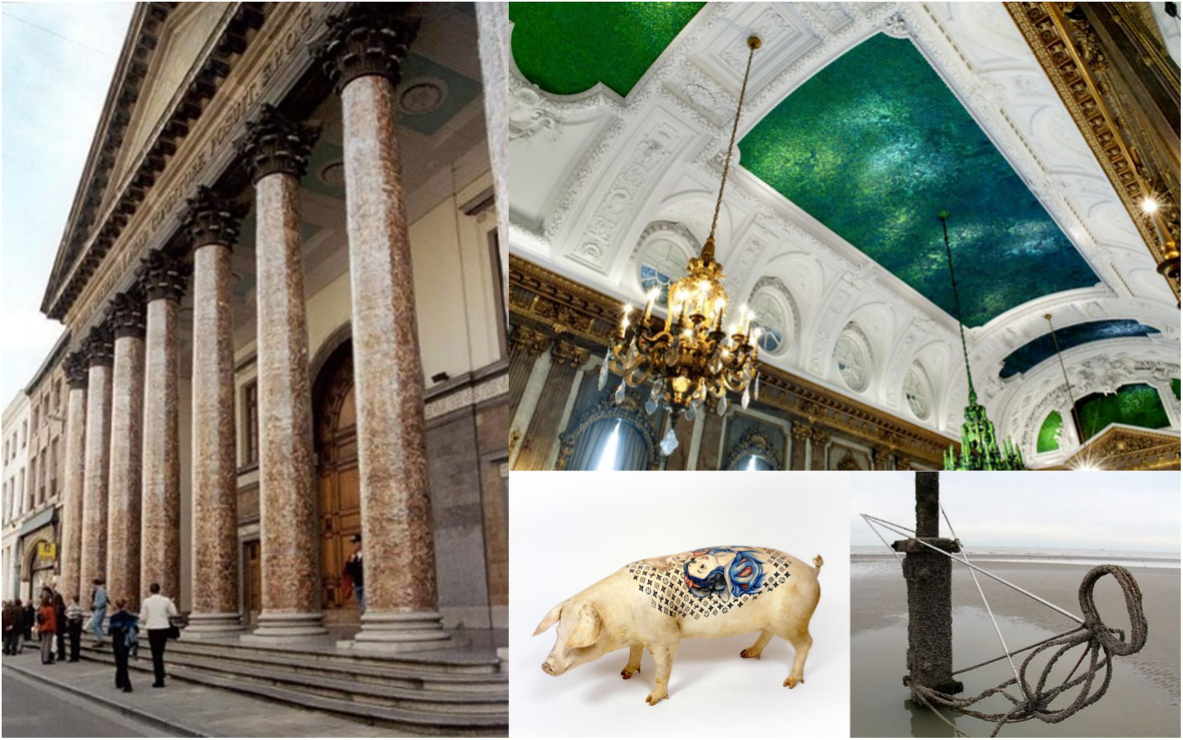
Belgian artwork made with or from animals. On the left, pig ham wrapped around the pillars of the UGent Aula building by Jan Fabre. On the top right, a beetle-covered ceiling in the Royal Palace in Brussels (Jan Fabre). On the bottom right, tattoed, living pigs by Wim Delvoye and “Beaufort-Art,” molluscs and shellfish growing on metal constructions in the tidal zone of the North Sea.

Other recent and strange examples are the slices of pig ham, glued on and covering completely the eight Corinthian pillars of the 200-year-old classical Ghent University main Aula building, and the jewel scarab beetle (*Chrisina gloriosa; Sternocera* sp.) green wings/bodies that covered and decorate since 2002 the ceiling in a room of the Royal Palace in Brussels, Belgium. Both artistic realizations are from controversial and now disgraced Belgian artist Jan Fabre (°1958). Another example is the tattooed live pigs of Belgian Wim Delvoye (°1965). Though both artists were not the first to make art, based on interactions with (parts of) animals (mammals and insects) (Fig. 5). All along the Belgian North Sea coast, from 2003 onwards till today, the “Beaufort-Art” series displayed *in vivo* artworks on the beach and in the tidal zone (Fig. 5): On the official beach poles metal constructions were fixed, which in function of ebb and flood tide were gradually overgrown—upon adhering with their protein-based glue—by shellfish (barnacles, *Sessilia* sp.) and mollusca (mussels, oysters, etc.) in a period of weeks to months. This finally ended up in a living piece of art and attracted lots of attention of tourists and artists. It raises the question: “Who is here the artist?”. And are wasp nests (cellulosic), honeycombs (waxy), and termite mounds then not arty constructions just because they are made by insects?

### Derived From Microbes

**Fig. 6.**
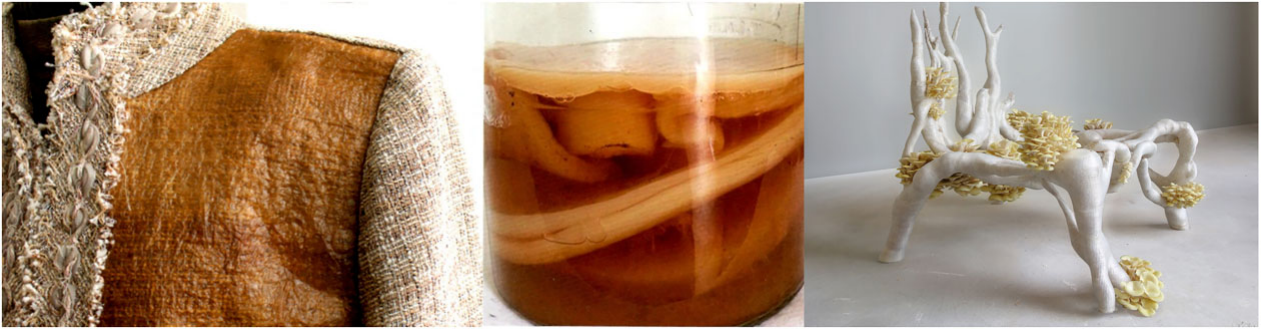
From left to right, bacterial cellulose used as bio-fiber in clothing, kombucha tea cellulose film, and a mycelium-based chair 3D-printed by Dutch designer Eric Klarenbeek.

Bacterial polymers (such as cellulosic fibers and films/sheets), naturally produced by acetic acid bacteria via surface and submerged fermentation processes, are nowadays “en vogue.” However, bacterial cellulose fibers are being formed spontaneously since ages during artisanal brewing of kombucha tea and “nata” drinks (Fig. 6), and are based on this natural activity of especially a range of acetic acid bacterial species such as *Acetobacter (Gluconacetobacter) xylinus* sp., *Komagataeibacter xylinus, Azotobacter* sp., and others (Avcioglu et al., 2021; Brown, 1886, 1996; Digel et al., 2023; Gorgieva & Trcek, 2019; Heydorn et al., 2023; Li et al., 2021; Sperotto et al., 2021; Vandamme et al., 1998; Walker et al., 2023, 2024; Zhong, 2020). In addition to food applications (Shi et al., 2014; Azeredo et al., 2019) and personal care and medical applications (Carvalho et al., 2019), bacterial cellulose is in use as bio-fiber for medical and technical application (Chen et al., 2018; Zhong, 2020) and for high-fashion luxury clothing (Fig. 6), furniture, technical textile (raw material for plant-free rayon, viscose and fabric, etc.), composite materials, or to construct canvases for paintings, drawings, etc. (Bianchet et al. 2020; Huang et al. 2014; Iguchi et al., 2000; Kesh, 2014; Reinati et al., 2017; Romling & Galperin, 2015). Recently, engineered strains of *Komatagaeibacter rhaeticus* have been constructed that produce “self-dyeing” melanin cellulose by expressing tyrosinase (Goosens et al., 2021; Walker et al., 2023, 2024) and strains of *Gluconacetobacter hansenii* excreting a cellulose-composite nanofibril of cellulose and hyaluronan (Takahama et al., 2021). Also the network of hyphae filaments (named mycelium, with chitin as a main component) of filamentous fungi, such as *Fusarium* sp., *Aspergillus* sp., and *Penicillium* sp., is intentionally grown by surface fermentation as a skin/membrane or by submerged fermentation on sugary substrates. *Fusarium venenatum* sp. mycelium is consumed as a vegetarian food supplement (Quorn®). It is marketed as a meat replacer in the U.K. and Europe since the 1990s. These and other fungal hyphae are now also co-woven into conventional fibers or plastics and they are in use for art furniture, bio-dresses, high-fashion clothes, handbags, fabric, artistic lampshades, etc. (Fig. 6) (Wood, 2017).

## Colorants Derived From Plants, Animals, and Microbes, and Their Use in Works of Art Over Time

### Colors, Dyes, and Pigments: Derived From Nature, Chemical Synthesis, or Fermentation and Microbial Biotechnology

#### *Chemicals* versus biochemicals

Colorants (dyes and pigments) are (bio-)chemicals that function by absorbing varying amounts of light at different wavelengths of the visible spectrum (wavelength ±400 to 750 nm), transmitting (if translucent) or reflecting the remaining light in straight or scattered lines (Diehl, 2008). Their constituent compounds may be classified chemically as inorganic (often from a mineral source) and organic (often from a biological source). Inorganic colors, such as in minerals, have been used as coloring agents since antiquity such as red ochre (Fe_2_O_3_), scarlet cinnabar (HgS), and orange–red realgar (As_4_S_4_) (Fig. 1). Colorants can be used for many purposes including printing, painting, and coloring or decorating many types of materials such as wood, stones, ceramics, plastics, chemicals, inks, clothing, fabric, but also cosmetics, food and feed, etc. and any other material. With the first synthetic dye preparation of mauveine in 1856 and the subsequent chemical preparation of thousands of other dyes, the importance of natural biocolors waned, but recently the tide is turning (Sousa et al., 2008). A number of chemically prepared colors such as azo colors plus aromatic amines and their degradation products such as benzidine-analogue colors have been proven to be actually toxic, carcinogenic, and difficult to decolorize and (bio)degrade (An et al., 2002; Chen et al., 1999; O’Neill et al., 1999; Solis et al, 2012; Sun et al., 2017). Currently, synthetic colors are being increasingly replaced in diverse applications by their biological (derived from plants, animals, or microorganisms) counterparts (Table 1). Many microorganisms such as bacteria, yeasts, fungi, and microalgae are known to be able to produce various colorants including phenazin, prodigiosin, violacein, carotenoids, porphyrin, chrysogenin, pulcherrimin, melanin, etc., but they have yet to be thoroughly examined for the possibilities of improved production and applications in diverse fields including works of art (Albermann & Beuttler, 2016; Babitha, 2009; Kim et al. 1999; Oshima et al. 1981; Wang et al., 2021). The fact that these colorants are of biological origin is clearly beneficial now that producer companies as well as the consumers are increasingly aware of health and environmental issues related to the use of chemically synthesized colorants. At the moment, the price tag of biocolorants is still too high for widespread use. In the near future, agricultural, biological, and biotechnological research in the fields of plant breeding, plant cell culture, and rDNA microbial fermentation processes will contribute to a broader application of these biocolorants not only in the food, cosmetic, pharmaceutical, technical, textile, and household sectors but also in the art world, craft, and design sectors (Rai et al., 2021; Reade, 2023; Wang et al., 2021; Yang et al., 2021).

**Table 1.** Examples of Synthetic and Biocolorants

Color	Synthetic Color		Biocolor		
	Name (E-nr.)	Application	Origin	Name (E-nr.)	Application
Black	Brillian Black (E1511)	F, P	Carbonized plants	Active carbon (E153)	F, C, P
Blue/purple	Patent Blue (E131); Indigo-Carmine (Indigotin) (E132); Brilliant-blue (E133)	C, P, T F, P	Grape peels, flowers, red cabbage Plants, rDNA—*E. coli* *Arthrospira*—cyanobacteria	Anthocyanin (E163) (Enocyanin) Indigo Phycocyanin	F, C T F
Green	Mixtures (E104 + E131, E104 + E132); Brilliant-green	F	Various plant extracts (spinach, nettles, alfalfa, and grass)	Chlorophyl (E140), Copper-chlorophyllin	F, C
Bright yellow	Tartrazin (E102), Chinolin-yellow (E104)	F, P	*Curcuma longa* root extract Maize, marigold, tagetes, microalgae Maize, *Flavobacterium* *Ashbya gossypii* fungus	Curcuma (E100), curcumine Lutein (E161b) Zeaxanthin Riboflavin or vitamin B_2_ (E101)	F, C A A F, A, C, P
Yellow (to red)	Sunet-yellow (E110) β-Carotene (E160a)	F, P F, P, C	*Bixa orellana* plant Saffronplant (*Croccus sativa*) Carrots, *Blakeslear trispora* fungus *Dunaliella* microalgae	Annatto (106b) (bixin, norbixin) Crocetine β-Carotene (E160a) and Xanthophylls (161a–f)	F, A F, C, P F, C, P
Orange (to red)	Orange yellow (E110)	F, P	Crustaceans, bird feathers, oilpalm, fungi, bacteria Plants Paprika plant extract (*Capsicum annuum*) *Adonis annua* flowers, *Xanthophyllomyces dendrorhous* yeast, *Haematoccus* microalgae	Canthaxanthin (E161g) Apocarotenal (E160e, f) Capsanthin, capsorubin (E160c) Astaxanthin	F, A F, P F, A A
Brown	Mixtures (E110 + E122 + E132), Brown FK (E154), Brown HT (E155)	F	Vegetable sugars	Caramel (E150)	F
Red	Litholrubine-BK (E180) Carmoisine (azorubine) (E122) Erythrosin (E127) Allura-red (E129), Amaranth (E123), Ponceau 4R (E124)	(F) F P, (F) (F), P	Scale insects *Monascus* fungi Tomato, watermelon Maize Red beet extract (*Beta vulgaris* L.) Grape peels *Lithospermum* plant cell culture	Cochineal (E120) (Carmine acid, Carmine, etc.) Monascin Lycopene (E160d) Morado Betalaines (E162) (betacyanin, vulgo xanthin) Enocyanin (E163) Shikonin	F, C, P F, C F, C, P F, A F, C F, P C, P

*Note*. A = (animal) feed; C = cosmetics; E-number = EU food additive legislation/directives; F = food; P = pharmacy; T = textile.

#### Colorants from antiquity till today

**Fig. 7.**
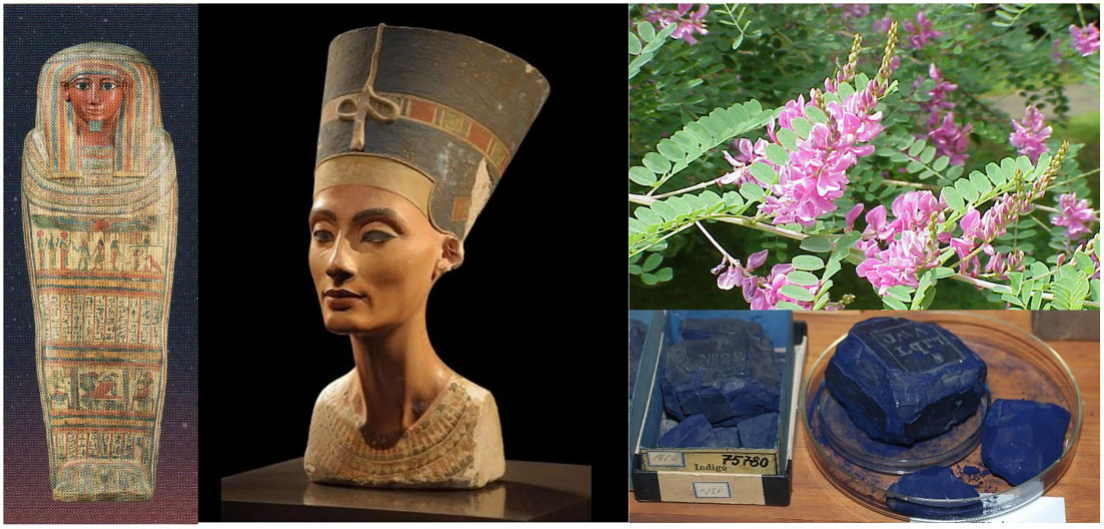
Egyptian use of biocolorants to decorate sarcophagi and statues (left) through the use of minerals and biopigments extracted from for example *Indigofera tinctoria* to obtain indigo dye (right).

Colorants play a very important but often underestimated role in human lives. Colors possess an esthetic and attractive value, they have a signal function and they can accentuate desired characteristics or mask deficiencies, and they do this since centuries! For example, since antiquity lichens, plants and insects have been used as sources of organic colors, suitable for multiple applications including textiles, clothing, cosmetics, food, paints, and works of art (Finlay, 2002; Grewal et al., 2022). Primitive depictions of humans, animals, and plants were already made by our ancestors since before 37,000 bc. Well-known early examples are rock and cave petroglyphs (carvings) and paintings. Examples are those in Australian Gabarnmang (28,000 bc) and in the Chauvet (37,000 bc) and Lascaux (17,000 bc) caves in France (Fig. 1): The colors used are of mineral and/or plant origin. Also, the robes and sarcophaguses of Egyptian mummies (ca. 3,600 bc) are ingeniously made, adapted, and colored. Ancient Egyptians used wood, plant resins, and plant fibers as biomaterials, in combination with black soot and charcoal (carbonized wood), yellow–greenish lichens (symbiosis of a fungus and microalgae or cyanobacteria), and plant extracts such as indigoblue as biopigments (Fig. 7). The colorful makeup of Egyptian queen Nefertiti (ca. 1370–1330 bc) still fires everyone’s imagination! In his “Historia Naturalis,” Pliny the Elder (ad 23–79) mentioned the coloring of young red wines with the juice of beetroot (*Beta vulgaris* L.) so that they would look older and pricier. French king Louis XIV (1638–1715) used to punish such practices, even with the death penalty. The red beet color (betanin) is now frequently used to color foods and fruit yoghurt (Piccaglia & Venturi, 1998). The abovementioned evolution has reintroduced the interest and the use of natural colors of biological origin. In addition, over the last decades, a lot of attention has been paid to the biotechnological synthesis of colorants through microorganisms (Albermann, 2011; Babitha, 2009; Dean, 1992; Jones, 1993; Nadogawithana, 1998; Yang et al., 2021). Current genetic or metabolic engineering techniques of colorant-producing microbial strains can lead to economical production of colorants on a larger scale through fermentation of renewable agro-industrial waste substrates next to starch, molasses, glucose, or sucrose (Grewal et al., 2022; Lee & Schmidt-Dannert, 2002; Murdock et al., 1993; Nishizaki et al., 2007; Vandamme, 2002; Wick, 1995; Yoshida et al., 2009). These biocolors are also easily and completely biodegradable after their use, contrary to many chemical dyes (Solis et al., 2012). Following is an overview of the ancient and contemporary bio-preparations and applications of some important natural biocolors in daily life and in works of art.

### Violet, Blue, and Purple Biocolors (400–490 nm)

#### Derived from plants

**Fig. 8.**
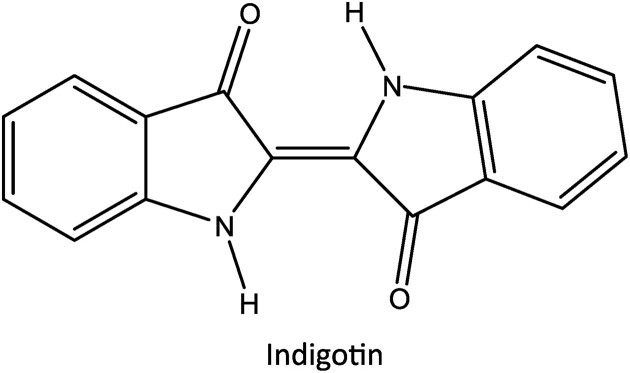
Chemical structure of indigotin, also known as “jeans blue.”

Indigo (E132) is one of the oldest known natural blue colors. Since approximately 1500 bc, Egyptian mummies were wrapped in garbs dyed with indigo. In Europe, indigo was initially obtained indirectly from the woad plant (*Isatis tinctoria*) and from the 16th century onwards from the tropical indigo plant (*Indigofera tinctoria*). In China, Japan, and South-Eastern Asia, indigo is derived from Dyer’s knotweed (*Polygonum tinctorium*) (Fig. 7). The green leaves of these plants contain the colorless indigo precursors isatan A and B and indican. Spontaneous fermentation of freshly picked indigo plant leaves by natural microbial flora yields—in a following steeping and chemical reduction process, called “vatting”—the yellowish water-soluble indigo white: a consortium of bacteria such as *Amphibacillus* sp., *Bacillus alkaliphilus, Clostridium isatidis*, and *Virgibacillus* sp. plays an important role in this natural process. Recently, axenic anaerobic fermentation processes for vatting of woad powder with *C. isatidis* and *Sinorhizobium meliloti* have been tried out (Kim et al., 2010; Milanovic et al., 2017; Osimani et al. 2012). Indigo is very important in textile dyeing and in the restoration of ancient paintings and tapestries. Fabric and textiles are then manually submerged in this indigo white solution. When the tissue is then removed from this bath, it is gradually transformed through oxidation in the air to the water-soluble indigo blue. Chemical or microbial reduction of indigo blue can again form indigo white. Such an indigo white solution is used to soak jeans cotton. Removal of the cloth from the dye bath permits oxidation with the air resulting in a slow transformation from yellow to green to “jeans blue” or indigotin (Fig. 8). This old technology is currently still widely used in rural areas in several countries in South-East Asia. In 1870, the chemical formula of indigo became known and attempts for its chemical synthesis started and were successful in 1896. From the 1920s onward, indigo blue has been chemically synthesized on a larger scale utilizing aniline, formaldehyde, and sodium cyanide, not exactly a green process. This synthetic indigo has nevertheless supplanted the natural indigo almost completely and is known universally as “jeans blue.” Stonewashed jeans are produced by washing the indigo-colored cotton cloth in drums filled with pumice. This is an expensive and laborious process. A “bio-stoning” process, developed by Novozymes (Denmark), uses a microbial cellulase to obtain the stonewashed effect more elegantly. In an alternative process, bacteria have been isolated that can degrade indigo. Their application can also lead to the well-known spotted and shabby look of the stonewashed jeans. There have also been attempts to grow cotton, crossed with wild, colored variants or through transgenic techniques, which would be naturally colored green, red, or blue in the field (Berry et al., 2002).

**Fig. 9.**
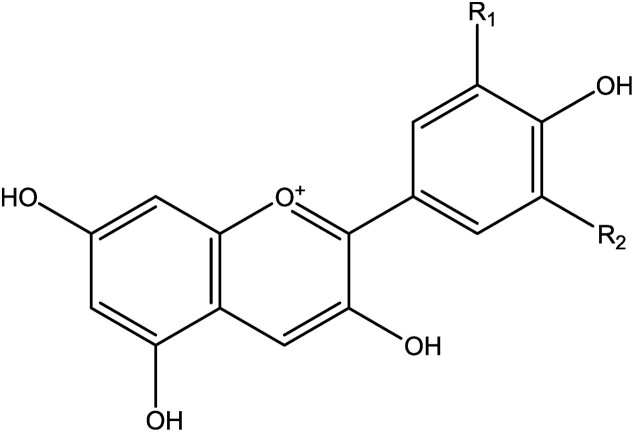
Chemical structure of anthocyanins delphinidin (R_1_= OH, R_2_ = OH), petunidin (R_1_ = OCH_3_, R_2_ = OH), and malvidin (R_1_ = OCH_3_, R_2_ = OCH_3_).

Purple and blue–greenish colors used to be produced also from lichens (Muggia et al., 2009) or extracted from tropical trees of the *Caesalpinaceae*. As these colors are not very colorfast, they were mainly used to illuminate old manuscripts because these remained largely unexposed to light. Everyone knows anthocyanins (Fig. 9) indirectly because of the splendid purple to red–brown autumn colors of the leaves of trees. In combination with the yellow–red carotenoids, they give leaves, flowers, and fruits a rainbow of colors. While they mainly occur in plants as glycosides, bound with sugar, the sugar-free compounds are called the anthocyanidins. The natural anthocyanins are now exclusively used as food colors, but in older times they were used in paints for precious manuscripts. They are now prepared from the peels of grapes (enocyanin) as a side product of the grape harvest and winemaking. Also red cabbage is a good source of anthocyanins (Piccaglia & Venturi, 1998).

#### Derived from animals

In the animal kingdom, *Murex* (or *Bolinus*) *brandaris* (a gastropod-sea snail of the *Muricidae*) also yields a nonfading purple dye called Tyrian or Imperial purple. It is a 6,6′-dibromo indigo compound in use for fabric dyeing since 1600 bc. It was the color of the robes of the wealthy, the royalty and of powerful religious and political leaders, etc. up till today and was also used in paintings (Ziderman, 1986).

#### Derived from microbes

Phycocyanin, a blue–green color that chemically consists of a protein coupled to chromophore tetrapyrrole groups, is a component of the photosynthetic pigment of cyanobacteria (*Nostoc* sp., *Arthrospira, Anabaena*, etc.) and microalgae. It is commercially produced based on the cyanobacterium *Arthrospira (Spirulina) platensis* and is used in human food in Japan (Francis, 1987; Martelli et al., 2014; Querques et al., 2015). The salt-loving red microalga *Porphyridium cruentum* is a source of the red–violet phycoerythrin that is structurally similar to phycocyanin. Such biocolors from microalgae are produced among others in Israel for cosmetic, fashion, and fabric applications (Borowitzka, 1992; Borowitzka & Borowitzka, 1988; Vandamme, 1989). The phycocyanin pigment has also been produced by genetically engineered *Escherichia coli* strains (Ge et al., 2013), and the search for novel halo- and thermotolerant high-producer cyanobacterial strains goes on (Bounnit et al., 2022). Since the 1990s, indigoblue, originally and still obtained from the indigo plant, can also be produced biotechnologically by bacteria starting from glucose. Several bacteria (*E. coli* and *Serratia* sp.) can produce the amino acid l-tryptophan from glucose through well-known biochemical pathways. l-Tryptophan is subsequently hydrolyzed into indol by the enzyme tryptophanase. The transfer of a *Pseudomonas* gene that encodes for naphthalene dioxygenase into *E. coli* results in a recombinant *E. coli* strain that is able to convert the substrate l-tryptophan, via indol, into an intermediary, which then spontaneously oxidizes into indigo (Murdock et al. 1993; Wick 1995). Bio-indigo has also been produced with a yield of 46.9% on a 3,000-L scale using rDNA *E. coli* strains harboring a flavin monooxygenase gene, with l-tryptophan as a substrate (Han et al., 2011). However, the current yield of bacterial indigo from glucose as a substrate has not yet resulted in an economically attractive bioprocess (Berry et al, 2002; Han et al., 2011; Rai et al., 2021; Reade, 2023; Vandamme, 2002, 2011; Vandamme & Revuelta, 2016). Pigments of *Chromobacterium violaceum* and of *Janthinobacterium lividum* such as violacein (navy blue) and deoxyviolacein (purple) have recently been overproduced with genetically engineered *E. coli* strains (Bisht et al., 2020; Cassarini et al., 2021; Yang et al., 2021). They can be applied in sunscreen additives and in dyes for fabric and artwork. Another bright blue natural dye, named indigoidine, was first isolated from phytopathogenic *Erwinia* sp. (Starr et al., 1966) and later from *Streptomyces chromofuscus* and *Photorhabdus luminescens*. Recently, it has been produced efficiently (over 8.0 g·L^−1^) by engineered *E. coli* strains (Xu et al., 2015) and could find use as a redox-state sensor, as an industrial dye, in fabric and artworks.

### Green–Yellow to Orange–Red Colors (490–620 nm)

#### Derived from plants

**Fig. 10.**
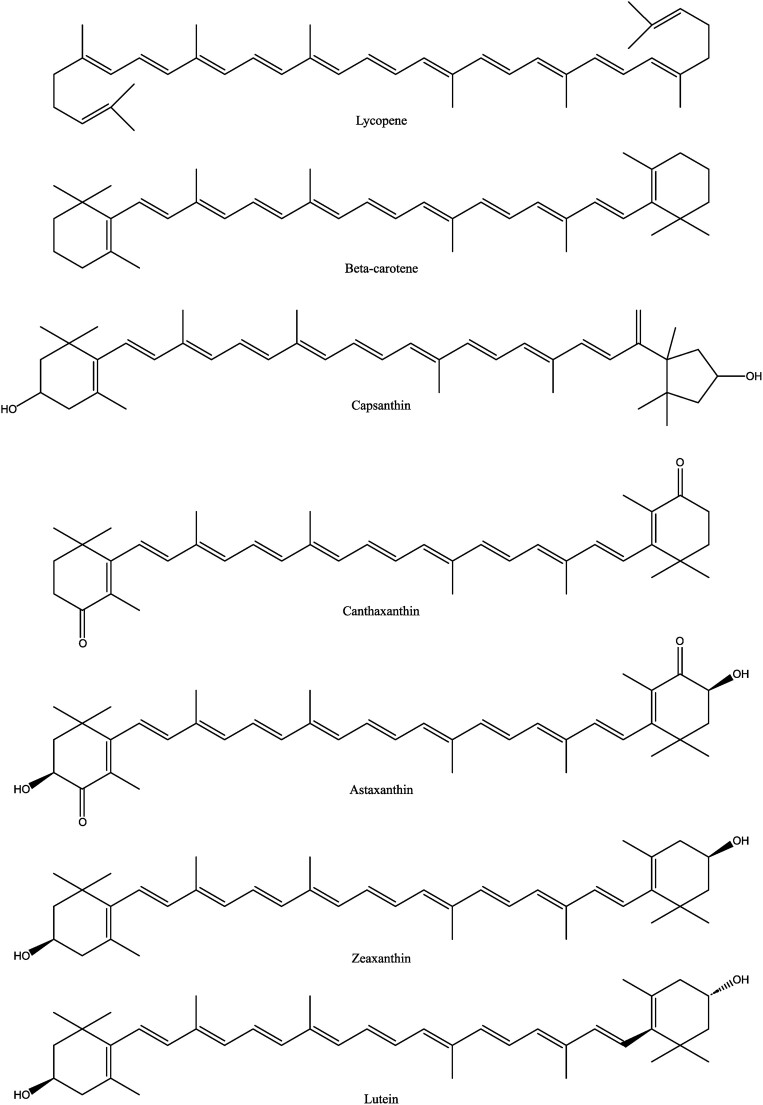
Chemical structure of various carotenoids.

The well-known yellow to orange biocolors include the flavones (e.g., luteolin) from the European yellow dyer’s weed or weld plant (*Reseda luteola*) or from dyer’s greenweed (*Genista tinctoria*), flavonols (quercetin) from rhododendron, and narcissine from the marigold (*Calendula officinalis*). They were commonly used in the past in paintings and for the coloring of robes and textiles (Piccaglia & Venturi, 1998; Wouters 1994). The yellow or orange–red carotenoids include the common carotenes and the xanthophylls (oxygen-containing carotenes) (Fig. 10). Carotenoids are found in red and yellow as well as green vegetables such as carrots, spinach, bell peppers, and broccoli, in fruits such as mangoes, apricots, avocados, and peaches, and in flowers such as *Tagetes* species (African marigolds). Carrots, for instance, contain beta-carotene, lycopene, and diverse xanthophylls, while lycopene is the most important red color in tomatoes. *Tagetes* flowers are very rich in the yellow carotenoid lutein. The seeds of the *Bixa orellana* plant contain the carotenoid annatto-color that is widely used in dairy food. Capsanthin, a xanthophyll, is the most important color in red bell pepper (*Capsicum annuum*). Carotenoids can also be retrieved as by-products in the processing of oil palm fruits into palm oil. Interest is growing in recovering these colorants from these easy-to-farm vegetable crops and their residues and use as biocolorants in food, feed, and technical applications including artworks.

#### Derived from animals

Carotenoids also occur in animals and are responsible for the pink to red color of salmon and crustaceans and also of the feathers of flamingos and poultry. Of particular note are canthaxanthin and astaxanthin. They are (exclusively) accumulated through the food chain, especially through carotenoid-containing microalgae or feed with components such as bell pepper meals. Various carotenoids have been chemically synthesized since the 1950s, but they remain very expensive. Recently, microbiological synthesis has gained importance (Frengova & Beshkova, 2009; Nishizaki et al., 2007; Parajo et al., 1998).

#### Derived from microbes (bacteria, fungi, and yeast) and from microalgae

**Fig. 11.**
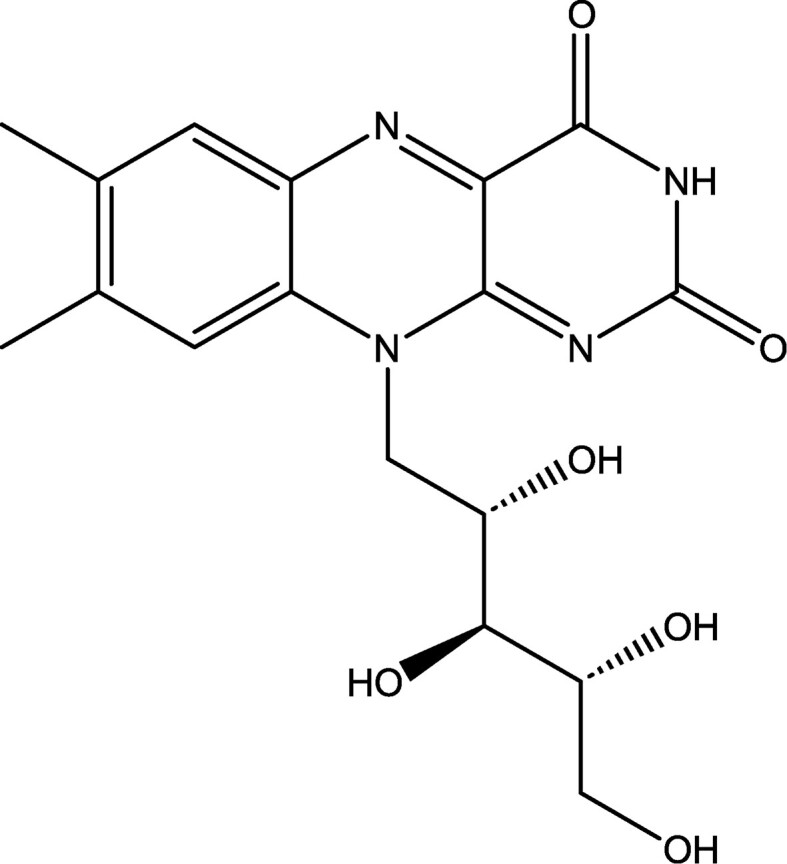
Chemical structure of riboflavin, often used as a yellow biocolorant.

Flavonoid compounds such as vitamin B_2_ (or riboflavin) (Fig. 11) display an intensely yellow color. Next to its function as a vitamin, riboflavin is increasingly used as a yellow biocolorant. Through fermentation processes with the fungus *Ashbya gossypii*, riboflavin is produced on a large scale (9,000 ton/year) by various companies in Europe, the USA, Japan, and China (Demain, 1972; Lago & Kaplan, 1981; Vandamme, 1989; Vandamme & Revuelta, 2016). Another commercial bioprocess (Takeda Chem. Ind., Japan) is based on the microbial synthesis of d-ribose from common sugars by *Bacillus subtilis* transketolase-negative mutants with d-ribose then chemically converted into riboflavin (Dewulf & Vandamme, 1997; Sasajima & Yoneda, 1971). Orange–red carotenoid compounds such as beta-carotene, astaxanthin, and others are also derived from microbes. Pure beta-carotene is used as a vitamin A precursor and as an antioxidant as well as an orange–red pigment in the food industry, pharmaceuticals, and cosmetics. It also possesses antitumor/anticancer characteristics. In the 1980s, a commercial bioprocess was developed on the basis of the controlled cultivation of salt-loving green microalgae (*Dunaliella salina, D. bardawil*) in basins, salt lakes, and lagoons. This is practiced in areas with favorable climatological conditions (sufficient sunlight, high temperatures, and a 20%–30% NaCl content) in Australia, Chile, China, Israel, Mexico, Spain, Russia, the United States, and South Africa. The harvested algal cells contain up to 14% beta-carotene in the dry mass with side products such as glycerol and protein-rich algal powder (Borowitzka & Borowitzka, 1988; Hejazi & Wijffels, 2004; Vorst et al., 1994). Another microbial process is based on the culture of the *Blakeslea trispora* fungus in large fermentors. Mixed cultures of the two sexual forms (mating type + and –) of this fungus produce up to 10 times more beta-carotene as a consequence of the induction of trisporic acid, a hormone that stimulates the beta-carotene biosynthesis. This process is industrially applied for the production of purified beta-carotene as well as production of beta-carotene-rich fungal mycelium that is used in animal feed. A cheaper process is based on the use—as a substrate—of fruits and vegetable waste in solid-state fermentation (Kaur et al., 2019; Nelis & Deleenheer, 1991; Ninet et al., 1969; Panesar et al., 2015; Prieto et al., 2011; Wang et al., 2014).

Astaxanthin (3,3′-dihydroxy-4,4′-diketo-beta-carotene) is the pigment that, through the natural food chain, turns salmon, trout, certain crustaceans, and the feathers of fowl pink. When those fish, crustaceans, and birds are reared, compounds such as astaxanthin are added to the feed in order for the pink color to develop. Synthetic astaxanthin and canthaxanthin (4,4′-diketo-beta-carotene) are frequently used. Related colors such as lutein and zeaxanthin (from maize) are also employed in the feed of fish and poultry. There is however a strong tendency to produce these colors through microbiological (bacterial, yeast, and microalgal) processes as many bacteria, yeasts, and microalgae synthesize carotenoids naturally. Over the years, several *E. coli* and *Saccharomyces cerevisiae* strains have been genetically modified to hyperproduce carotenoids (Albermann & Beuttler, 2016; Grama et al., 2016; Guo et al., 2018; Jiwara et al., 1995; Li et al., 2020; Mata-Gomez et al., 2014; Miura et al., 1998; Mussagy et al., 2019; Olson et al., 2016; Sandmann, 2015; Stoklosa et al., 2019). Only a few are cultured on a large scale for pigment production. In this respect, DSM-Firmenich (the Netherlands) has devised a fermentation process in which the yeast *Xanthophyllomyces dendrorhous* (previously *Phaffia rhodozyma*) produces relatively large amounts of (3R, 3R′)-astaxanthin. A similar process has been developed by Sensient Technologies Corp. (USA) and Igene (USA) that market an astaxanthin-rich yeast preparation. Astaxanthin has also been found in salt-loving bacteria, for example, *Halobacterium salinarum*. Microalgae, such as *Haematococcus* sp., are now also being cultured on a large scale in open ponds in the Atamaca Desert in Chili (Grama et al, 2016; Li et al., 2011; Nelis & Deleenheer, 1991). All the genes that code for astaxanthin biosynthesis had been identified and cloned in the host cell bacterium *E. coli* (Jiwara et al., 1995). Also, several carotenoids (lycopene, zeaxanthin, and xanthophylls) can now be produced via genetic modification of the food yeast *Candida utilis*, other yeast, and several bacterial species (Cheng, 2006; Johnson & Schroeder, 1995; Li et al., 2015; Miura et al., 1998; Sun et al., 2014; Zhu et al., 2015). If this will lead to a successful industrial fermentation process remains an open question as a result of the ongoing distrust of many consumers toward the use of Genetically Modified Organisms (GMOs) in the food and feed chain (Albermann, 2011; Albermann & Beuttler, 2016; Lee & Schmidt-Dannert, 2002; Nishizaki et al., 2007; Verwaal et al., 2007; Yoshida et al., 2009). The bacteria *Flavobacterium* sp. and *Pantoea* sp. are able to form naturally high amounts of the yellow maize pigment zeaxanthin (3,3′-dihydroxy-beta-carotene) (Nelis & Deleenheer, 1991). It can now also be produced via engineered *E. coli* strains (Li et al., 2015). Canthaxanthin is found in the edible mushroom *Cantharellus cinnabarinus*. It can also be produced with engineered *E. coli* strains (Scaife et al., 2012). These microbial carotenoids might be used as colorants in works of art if they could be produced cheaply in high yield.

### Red Biocolors (620–750 nm)

#### Derived from plants

**Fig. 12.**
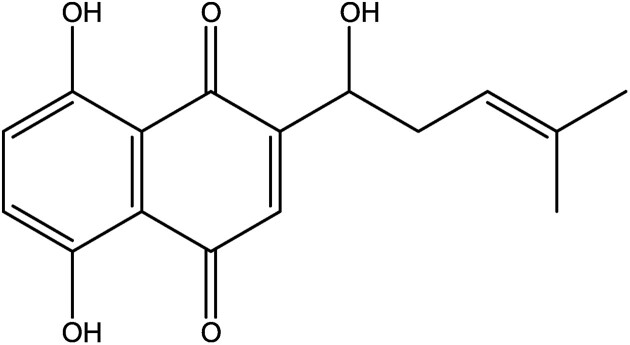
Chemical structures of the red biocolorants brazilin, alizarin, and shikonin (from left to right).

The ancient Egyptians knew by 4000 bc that the flowers of *Carthamus tinctorius* contained a valuable color, safflower, which is a mixture of safflower–yellow and safflower–red carthamin. Cloth dyed with this pink–red quinone color does not discolor. Natural quinone biocolors possess excellent characteristics, even according to modern standards. Some display a glow, light colorfastness and stability that has not yet been equaled by synthetic colors. This led to the planting, growing, and harvesting of the flower petals of *C. tinctorius* on a large scale in among others India, Far East, and Egypt, where it was used to dye silk and cotton fabric. It was also used to dye the red cotton tapes of legal documents and is at the origin of the expression “red tape” (Piccaglia & Venturi, 1998). Plants such as Brazilian redwood, red cabbage, red beet (*Beta vulgaris*), and madder (*Rubia tinctorum*) were also a valuable source of red colorants (respectively brazilin, anthocyanin, betalains, and alizarine). These were used in art painting and to dye and illustrate costly manuscripts, hanging wall tapestry, robes, and art furniture. Several of these plants were cultivated since medieval times as colorant crops, also in Western Europe. Brazilin (Fig. 12) is a red dye prepared from the wood of tropical trees such as *Paubrasilia echinata, Caesalpinia violacea*, and *Biancaea sappan* that was used in ink, paints, and to dye fabric. Red cabbage (*Brassica oleracea*) is rich in red acylated anthocyanin pigments and has more stable colors, contrary to nonacylated red grape pigments. Betalains from red beet are now mainly used as food colorants (Socaciu, 2008). The bright red quinone color alizarin (Fig. 12) is found in the roots of various plants but especially in *Rubia tinctorum* L., the madder. The extracted alizarin was widely used for the painting of textiles and leather. It was a precious and desired textile dye, called “Turkish red,” that was cultivated in the provinces of Zeeland and Brabant in the Netherlands from the Middle Ages till the end of the 19th century. This bioproduction was halted when the synthetic colors were introduced, particularly when in 1868 alizarin could be prepared from coal tar. The cultivation of madder has recently been reintroduced in the Groningen province in the Netherlands in conjunction with the study of improved alizarin extraction and purification techniques. In the meantime, many synthetic anthraquinone colors have been prepared and applied, especially to produce violet, blue, or green hues in the textile sector. A lifecycle analysis of the alizarin color showed that synthetic (nature-identical) alizarin still has a better environmental profile (as to emissions and global energy consumption) than naturally produced alizarin. The introduction of improved plant breeding and extraction techniques can however turn the balance. In addition, fermentation processes to culture hairy root tissue and cells of madder to produce alizarin and others have been optimized (Masahiro et al., 1994; Yang et al., 2021). The plant naphthoquinone esters of shikonin (Fig. 12) and alkannin are red colorants that have been used for ages for dyeing wool, cotton, and silk. In earlier days these pigments had to be extracted from the roots of 5-year-old *Lithospermum erythrorhizon* plants (growing in China, Korea, and Japan) and *Alkanna tinctoria* plants (growing in arid maritime regions of the Mediterranean), which kept the price of shikonin and alkannin esters very high. Since 1983, it has been produced by Japanese company Mitsui Petrochemical Industries through submerged plant cell culture in bioreactors. It is employed for coloring luxury and artistic fabric and textiles and also as a skin balm in cosmetics such as lipstick and in pharmaceuticals as an inhibitor of hemorrhoid inflammations (De Leo et al., 1996; Fugita et al., 1985). Plant and microbial cell culture technology, combined with rDNA-technology, are now increasingly applied for *in vitro* colorant production of these (or new) colorants (Goosens et al., 2021; Grewal et al., 2022; Reade, 2023; Vandamme, 2002; Vandamme & Revuelta, 2016; Walker et al., 2023, 2024; Yang et al., 2021).

#### Derived from animals

**Fig. 13.**
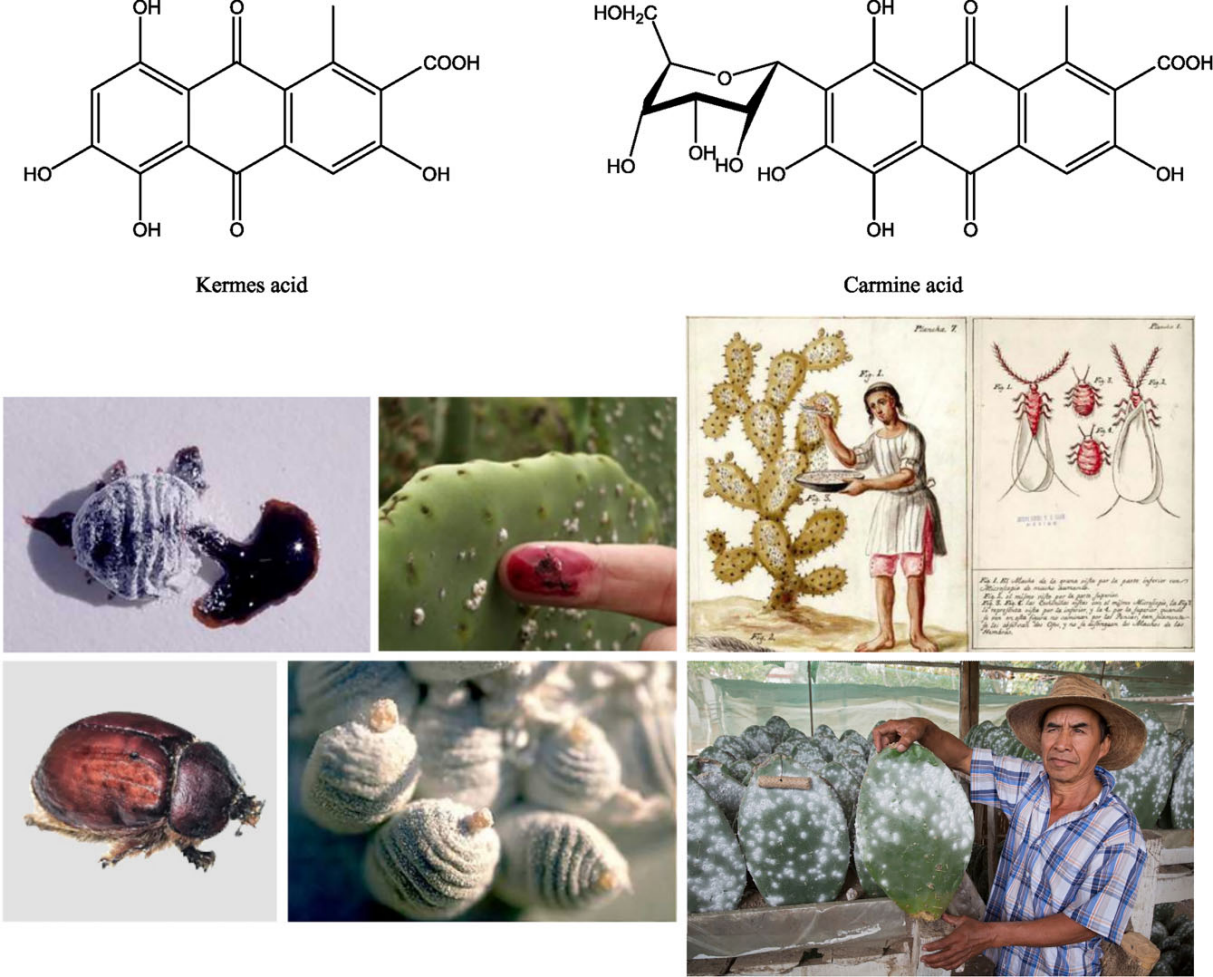
Top: Chemical structures of the red biocolorants kermes acid and carmine acid. Bottom: Harvesting of scale insects to obtain these red biocolorants.

Anthraquinone colors such as kermes (active substance kermes acid) and cochineal or scarlet (carminic acid, carmine, and crimson lake [E120]) (Fig. 13) are obtained from the scale insects *Kermes vermilio, K. biblicus* (living on the kermes oak), *Dactylopius coccus* (living on *Opuntia* cactus plants), and *Porphyrophora hamelii* and *P. polonica* (living on the roots of plants) (Fig. 13). Today, kermes color has only art history value in the analysis of antique works of art, paintings, robes, and tapestries. Cochineal is still produced on a relatively large scale (Greenfield, 2005). The scale insects are manually collected, especially from their cactus hosts, on plantations in countries such as Peru, Bolivia, Mexico, Chile, Argentina, and the Spanish Canary Islands. This process was already practiced by the Inca and Aztec cultures (ad 1200–1520). The carmine color is extracted from the insects and this expensive color is now still applied in exclusive cosmetics, paints, robes, and foods such as the Campari aperitive and cocktail cherries (Francis, 1987; Nagodawithana, 1998; Wouters 1994). Cochineal for instance possesses a glow/shine, light colorfastness and color stability that has not yet been equaled by synthetic colors. Famous Flemish painter Jan van Eyck (1390–1441) used it in his paintings and Cosimo the Elder De Medici is often depicted in a scarlet robe, only affordable by the superrich class and cardinals. It can recently be produced via biotechnology with rDNA *E. coli* bacteria (Yang et al., 2021).

#### Derived from microbes

**Fig. 14.**
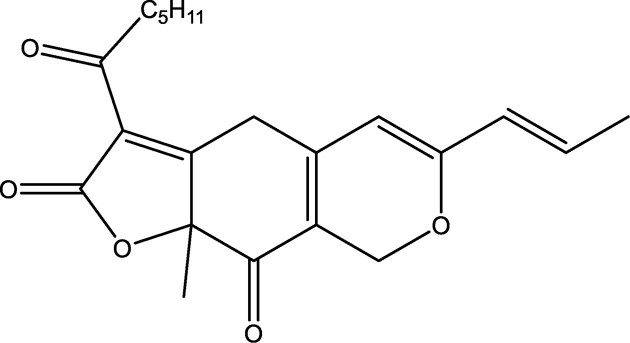
Chemical structure of monascin.

Red prodigiosins and analogues are known as colorants to be produced by a range of bacteria such as *Serratia marcescens, Rhodonellum psychrophylum*, and *Vibrio* species. They also display antimicrobial and antioxidant activity and are used to develop antimicrobial fabrics and to dye wool, silk, and synthetic fibers (Bisht et al., 2020; Vandamme, 2012; Venil et al., 2013). Anthraquinones colorants are also produced by many fungi and bacteria. Further research can lead to a fermentation process for the production of these interesting compounds. Monascin azaphilone (Fig. 14) polyketide pigments (MPs) varying from yellow over orange to red are a group of secondary metabolites produced by several *Monascus* fungal species. In the Far East, especially in China, Japan, Thailand, Indonesia, the Philippines, etc., they are used for over two millennia as a natural pigment and food colorant. The red colored monascin is produced *in situ* by active fungal cells among others in fermented ang kak (red yeast rice), red koji, hongqu, etc. or it can be added in surimi (fish paste) and pink saké wine and many other foods and drinks. This color is being produced by the fungal species *Monascus purpureus, M. ruber*, and *M. anka* especially through solid-state fermentation processes on moist rice. Monascin pigments consist of over 60 closely related polyketide structures that vary in color from bright yellow to deep red. It is a natural color that could replace the synthetic erythrosin (Red nr. 3 or E127). In addition to food applications, it could be used in cosmetics and to color textiles such as silk, leather, and linen and cotton fiber, and thus ends up in artwork and expensive fabrics, etc. (Shi et al., 2022; Yang et al., 2018). Polyketide pigments’ art history value relates so far mainly to the analysis of antique art, paintings, robes, and ancient tapestries. Polyketide pigments also display several physiological activities such as antimicrobial action, antioxidant, and enzyme inhibitory activity (Antipova et al., 2023; Feng et al., 2016; Juzlova et al., 1996; Patakova, 2013).

## Action of Microbes Displayed—Unknowingly—in Art

**Fig. 15.**
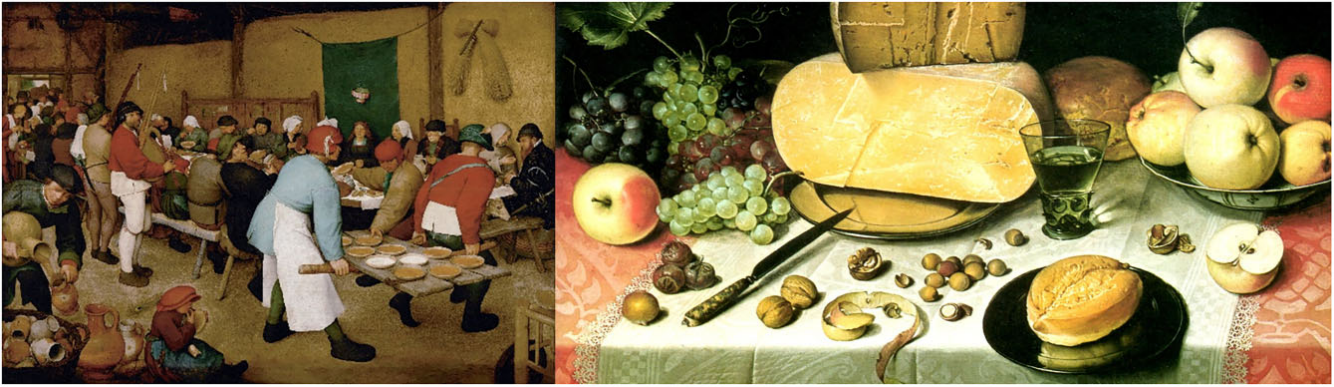
Renaissance painters Pieter Bruegel the Elder and Floris Claesz. Van Dyck depicting microbial activity (cheese, bread, and wine). Left: “The Peasant Weddings.” Right: “Still Life with Cheeses.”

Several famous painters have unknowingly depicted the activities of yeast among others the Flemish Renaissance painter Pieter Bruegel the Elder (ca. 1525–1569) in his famous painting of 1567 “The Peasant Wedding,” where serving beer, wine, and bread are depicted (Fig. 15, Left). Also bacteria and yeast were unknowingly depicted by Dutch painter Floris Claesz. van Dyck (ca. 1575–1651), in his painting “Still Life with Cheeses” of 1613; sliced cheese balls and loafs of bread decorate a richly set table (Fig. 15, right). Beer brewing and cheese making were at that time biotech processes “avant la lettre,” where— as we know now— yeasts, lactic bacteria, and enzymes fulfill an essential but invisible role. At that time, the existence of microbes and their essential role in fermented foods and drinks production was unknown, till the findings of Louis Pasteur (1822–1895) and his contemporaries in the period 1857–1880 forced a breakthrough (Vandamme, 2021a).

## Living Microbial Art

**Fig. 16.**
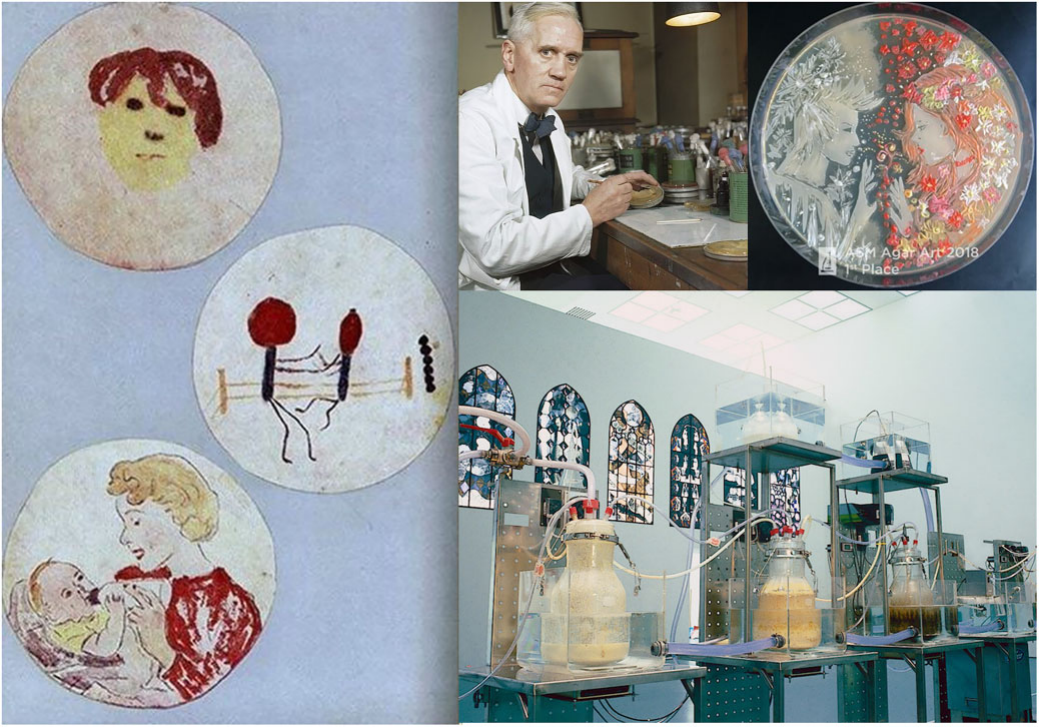
Left: Microbial art made by Sir Alexander Fleming. Right: Sir Alexander Fleming behind his desk creating agar art, the first place winning agar piece of the agar art competition of 2018 organized by the American Society of Microbiology, and on the bottom the “cloaca artwork” created by Wim Delvoye.

The use of living microbes as works of art was introduced by Sir Alexander Fleming (1881–1955), the discoverer of penicillin in 1928, while practicing his hobby, now called “germ painting, mold art, or microbial art” (Fig. 16). He allowed microbes to develop “paintings” in Petri dishes, by inoculating pigment-producing bacteria, yeasts, and fungi in a certain pattern on a layer of agar as canvas. The colorful piece of miniature art became visible upon incubation and outgrowth into colony form! This concept has now found many imitators/followers. The American Society of Microbiology, the U.K. Society for Applied Microbiology, the Belgian Society for Microbiology, etc. and several others select and present yearly awards for the best colorful and original “microbial paintings” (Fig. 16) (Brannon, 2013; Dixon, 2013a; Maloy, 2016; Wood, 2017). The controversial “cloaca,” an “art and poop-machine” devised by earlier mentioned Belgian artist Wim Delvoye, simulates the human physiological digestive process *in vitro* in the stomach, small, and large intestines, where mainly a range of bacteria are doing the job. In this “cloaca artwork,” a series of coupled bioreactors simulates this bioprocess, where bacteria have an essential role (Fig. 16). This artistic bioprocess is based on earlier findings from basic human physiological scientific research, on controlled fermentation technology and on fundamental knowledge about the actions of human intestinal bacteria. This principle and concept was well documented in scientific journals and is known as the “Shime reactor: *Simulation of Human Intestinal Microbial Ecosystem*” (Gibson & Rastall, 2004; Molly et al., 1993).

## Damage to Works of Art and Repair via Microbial Biotechnology-Based Techniques

In addition to physical and/or chemical deterioration and damage of artworks (frescos, statues, paintings, etc.) in musea, caves, tombs, churches, etc. caused by dust, moisture, light, weathering, corrosion, polluted air, vandalism, flooding, etc., also (micro)biological damage and deterioration occurs over time (Barton & Jurado, 2007; Ciferri, 1999; Dixon, 2005, 2013a, 2013b; Gonzalez et al., 2008; Konkol et al., 2008; Lopez-Miras et al., 2013; Pepe et al., 2010; Rahman et al., 2016). A range of bacteria, fungi, microalgae, insects, etc. affect colorants, pigments, varnish, glue, canvas, fibers, frames, etc. depending on indoor and outdoor environmental conditions. Rapid isolation, identification, and characterization of the involved (micro)-organisms rely now on a combination of conventional microbiology and molecular DNA-based techniques, often combined with chemical and physical analyses. Based on these results, appropriate biocides, fumigation, ozonation, and disinfectants, but also selected microbial strains can be applied, followed by restoration and conservation (Cappitelli et al., 2020; Konkol et al., 2008; May & Jones, 2006).

## Two Cultures …? Mind the Gap!

The foregoing examples indicate that—largely unknown by the public—the role and impact of biology, microbiology, and biotechnology in creating art over centuries is broader and far older than anticipated at first sight. In this context, it is strange that even today the sciences and the arts remain largely separated worlds, apart from exceptions as described earlier. Famous British scientist, novelist, and politician Charles Percy Snow (1905–1980) mentioned this discord already in 1959 in his book *The Two Cultures and the Scientific Revolution*, indicating that artists live on a “shoreless island” and scientists on a “diked island” (Snow, 1959). He argued that the lack and poor communication—the cultural gap—between the two cultures of modern society—the sciences and the humanities—was a major hindrance to solving the world’s problems. We all have to mind this gap! Indeed, interactions between these two separated worlds are urgently to be enforced and luckily initiatives to close this gap are emerging (Bouckaert, 2019; Monard, 2021; Schwanauer & Levitt, 1984; Vandamme, 2021b). Art serves as an important medium to communicate science and can inspire students to pursue STEAM (Science, Technology, Engineering, the Arts, and Mathematics) studies (Frankel et al., 2023).

## Data Availability

No new data were created or analyzed during this study. Data sharing is not applicable to this article.
